# Light‐dependent expression of a Na^+^/H^+^ exchanger 3‐like transporter in the ctenidium of the giant clam, *Tridacna squamosa*, can be related to increased H^+^ excretion during light‐enhanced calcification

**DOI:** 10.14814/phy2.13209

**Published:** 2017-04-24

**Authors:** Kum C. Hiong, Anh H. Cao‐Pham, Celine Y. L. Choo, Mel V. Boo, Wai P. Wong, Shit F. Chew, Yuen K. Ip

**Affiliations:** ^1^ Department of Biological SciencesNational University of SingaporeKent RidgeSingapore; ^2^ The Tropical Marine Science InstituteNational University of SingaporeKent RidgeSingapore; ^3^ Natural Sciences and Science EducationNational Institute of EducationNanyang Technological UniversityNanyang WalkSingapore

**Keywords:** Acid‐base balance, ammonia, mantle, proton, tridacnid, zooxanthellae

## Abstract

Na^+^/H^+^ exchangers (NHEs) regulate intracellular pH and ionic balance by mediating H^+^ efflux in exchange for Na^+^ uptake in a 1:1 stoichiometry. This study aimed to obtain from the ctenidium of the giant clam *Tridacna squamosa* (*TS*) the complete cDNA sequence of a *NHE3‐like transporter* (*TSNHE3*), and to determine the effect of light exposure on its mRNA expression level and protein abundance therein. The coding sequence of *TSNHE3* comprised 2886 bp, encoding 961 amino acids with an estimated molecular mass of 105.7 kDa. Immunofluorescence microscopy revealed that TSNHE3 was localized to the apical membrane of epithelial cells of the ctenidial filaments and the tertiary water channels. Particularly, the apical immunofluorescence of the ctenidial filaments was consistently stronger in the ctenidium of clams exposed to 12 h of light than those of the control kept in darkness. Indeed, light induced significant increases in the transcript level and protein abundance of *TSNHE3*/TSNHE3 in the ctenidium, indicating that the transcription and translation of *TSNHE3*/TSNHE3 were light‐dependent. As light‐enhanced calcification generates H^+^, the increased expression of *TSNHE3*/TSNHE3 in the ctenidium could be a response to augment H^+^ excretion in pursuance of whole‐body acid‐base balance during light exposure. These results signify that shell formation in giant clams requires the collaboration between the ctenidium, which is a respiratory and iono‐regulatory organ, and the inner mantle, which is directly involved in the calcification process, and provide new insights into the mechanisms of light‐enhanced calcification in giant clams.

## Introduction

**Figure 1:**
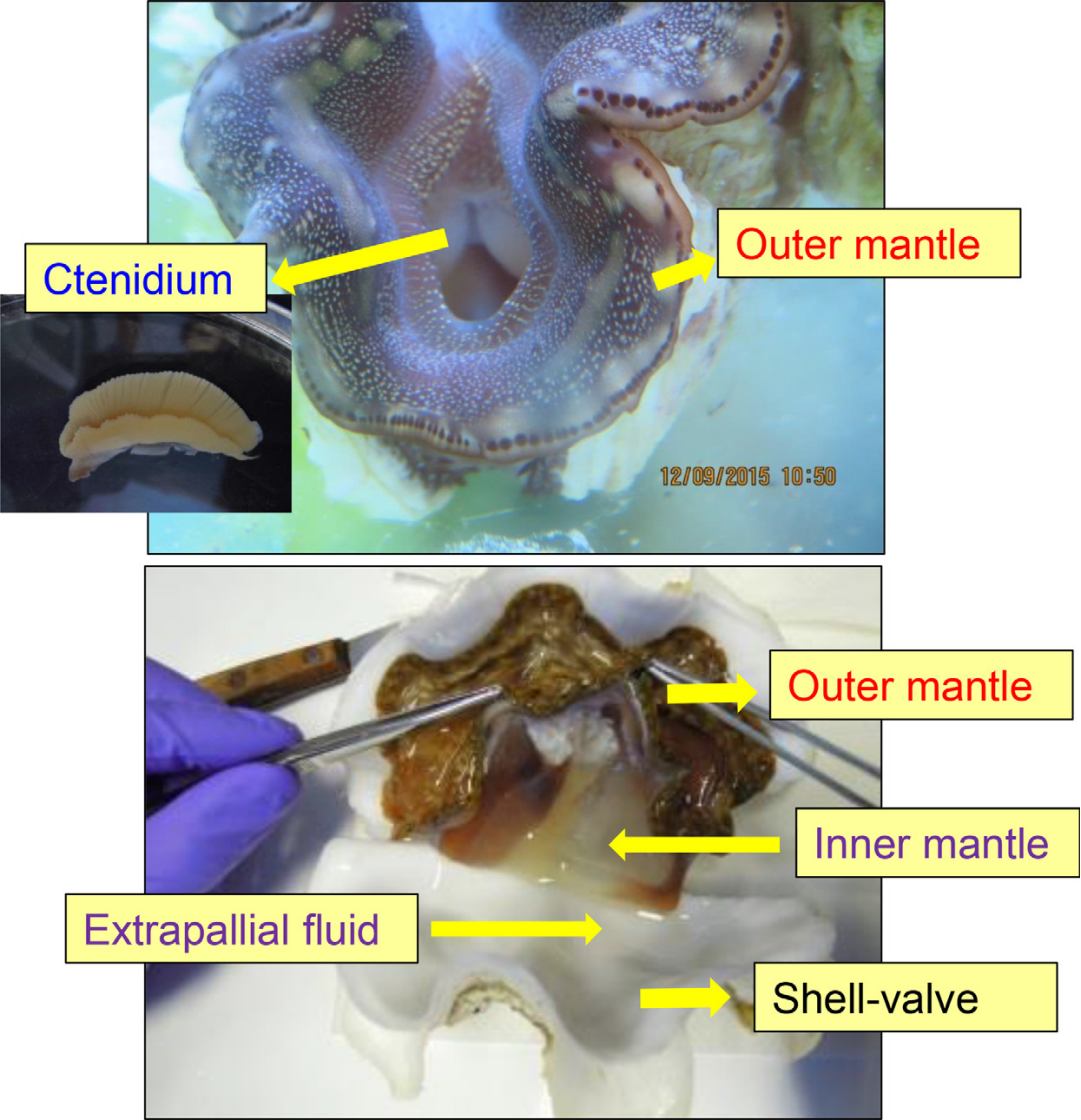
An illustration on the anatomy of the giant clam *Tridacna squamosa*. The ctenidium is a respiratory organ that takes part in ionoregulation and acid‐base balance. The extensible outer mantle harbors extracellular zooxanthellae in tertiary tubules and the inner mantle is in direct contact with the extrapallial fluid and it is involved in shell formation.

Giant clams (Family: Cardiidae, Subfamily: Tridacninae, Genus: *Tridacna*) are marine bivalve mollusks which can be found along coral reefs throughout the tropical Indo‐Pacific (Rosewater 1965). They harbor symbiotic zooxanthellae (*Symbiodinium*; Clade A, C and D) which reside extracellularly in a branched tubular system within their body. The tubular system originates from the stomach and splits into small secondary and tertiary tubes dorsally into the root of the siphonal mantle. The tertiary tubes are positioned under the surface of the extensible outer mantle (Norton et al. 1992), where the zooxanthellae can receive sufficient light for photosynthesis (Fig. 1). The outer mantle has iridophores which consist of small groups of cells (iridocytes) containing stacks of tiny flattened platelets (Griffiths et al. 1992). The reflective platelets scatter light of photosynthetically productive wavelengths into the tissue while back‐reflecting nonproductive wavelengths (Holt et al. 2014). Thus, the extensible outer mantle is brightly colored (Fig. 1). In contrast, the inner mantle adjacent to the extrapallial fluid within the pallial line is mostly nonpigmented (Fig. 1) and involved in shell formation (calcification). With the help of symbiotic zooxanthellae, giant clams can undergo light‐enhanced shell formation and maintain high growth rates in nutrient‐deficient marine environments (Lucas et al. 1989).

Shell formation in bivalves involves calcification, whereby calcium carbonate is deposited onto the inside surface of the shell‐valve according to the reaction: Ca^2+^ + HCO_3_^−^ ⇔ CaCO_3_ + H^+^. The removal of H^+^ leads to an increase in pH which would raise the supersaturation of aragonite and result in a more rapid precipitation of CaCO_3_. Indeed, exposure to light induces a significant increase in the pH of, and a significant decrease in the total ammonia concentration in, the extrapallial fluid of the fluted giant clam, *Tridacna squamosa* (Ip et al. 2006). Ip et al. (2006, 2015) proposed that, during light‐enhanced calcification, H^+^ released from CaCO_3_ deposition could react with NH_3_ to form NH_4_^+^ in the extrapallial fluid, and NH_4_^+^ could be transported subsequently into the shell‐facing epithelial cells of the inner mantle. There is also indirect evidence which suggests the involvement of a Ca^2+^‐ATPase in light‐enhanced calcification in *Tridacna derasa* (Sano et al. 2012). It is probable that this is a plasma membrane Ca^2+^‐ATPase which can act as an obligatory Ca^2+^/H^+^ exchanger (Salvador et al. 1988), transporting Ca^2+^ from the inner mantle epithelial cells to the extrapallial fluid and H^+^ in the opposite direction. Either way, the excess H^+^ entered into the shell‐facing mantle epithelial cells needs to be transported to the hemolymph and excreted elsewhere, so as to maintain cellular and whole‐body acid‐base balance. One possible site of H^+^ excretion and whole‐body acid‐base balance is the ctenidium (or gill) which, despite being far away from the site of calcification, has a large surface area for respiration and ion transport (Fig. 1).

A ctenidium is a respiratory organ which is found inside the mantle cavity of many mollusks, including bivalves, cephalopods and numerous aquatic gastropods. It is white in color and consists of two demibranches (dorsal and ventral). There is one pair of demibranches on each side of the byssal digestive mass and reproductive organ. Each ctenidium is shaped like a comb, with a central part from which many filaments protrude and line up in a row to increase the surface area for respiration. A ctenidium can also take part in ionoregulation and acid‐base balance in mollusks. In cephalopods, enzymes and transporters related to acid‐base balance, including carbonic anhydrase, Na^+^/K^+^‐ATPase, V‐type H^+^‐ATPase, Na^+^:HCO_3_^−^ cotransporter and Na^+^/H^+^ exchanger (NHE), are expressed in specialized ion‐transporting cells in the ctenidium, which can be the major site for acid‐base regulation (Hu et al. 2011, 2014).

NHEs belonging to the solute‐carrier 9 family are transmembrane proteins that regulate intracellular pH and ionic balance by mediating Na^+^/H^+^ exchange in a 1:1 stoichiometry (Fliegel and Dibrov 1996; Counillon and Pouyssegur 2000). There are 9 isoforms of NHE (NHE1–9), each with distinct tissue expression, cellular localization, and physiological functions in mammals (see Donowitz and Tse 2001 for a review). Based on their subcellular localization, NHE1–5 is classified as plasma membrane proteins. NHE6–9 is present in intracellular membranes of organelles such as the Golgi apparatus, although NHE8 is also expressed in the apical membrane of polarized epithelial cells. Mammalian NHE1 plays a key role in regulation of cell pH, volume, and proliferation, and has basolateral localization in epithelial cells. NHE2 and NHE3 mediate Na^+^ absorption and H^+^ secretion, and are localized to the apical membrane of renal cells. About 50% of the overall apical NHE activity is mediated by NHE3 in the proximal convoluted tubule of mice (Choi et al. 2000). Similar to mammalian kidneys, fish gills are the main excretory organs responsible for iono‐regulation and acid‐base balance. Branchial Nhe isoforms can contribute to H^+^ secretion in marine teleosts (Claiborne et al. 1999; Edwards et al. 2005) and Na^+^ absorption in some freshwater species (Hwang and Lee 2007).

At present, there is a dearth of information on the role of the ctenidium in acid‐base balance in bivalves in general, and on the role of the ctenidium in light‐enhanced calcification in giant clams. We speculated that the ctenidium of *T. squamosa* would express an NHE transporter to mediate H^+^ excretion and acid‐base balance. Furthermore, we postulated that such a transporter would be expressed in the apical membrane and hence would be NHE3‐like. Therefore, this study was undertaken to obtain from the ctenidium of *T. squamosa* (TS) the complete cDNA sequence of an *NHE3*‐like transporter (*TSNHE3*)*,* and to determine the effects of 3 h, 6 h or 12 h of light exposure on its mRNA expression level therein. Based on the deduced TSNHE3 sequence, a custom‐made anti‐TSNHE3 antibody was made to examine the effects of light exposure on its protein abundance in the ctenidium. Immunofluorescence microscopy was also performed to confirm the apical localization of TSNHE3 in the epithelial cells of ctenidial filaments and tertiary water channels. As *TSNHE3*/TSNHE3 could be involved in the excretion of excess H^+^ produced in the extrapallial fluid during light‐enhanced calcification, the hypothesis tested was that the gene and protein expression levels in the ctenidium of *T. squamosa* would be light‐dependent.

## Materials and Methods

### Experimental animals

Adult specimens of *T. squamosa* (521 ± 184 g; *N *=* *30) were obtained from Xanh Tuoi Tropical Fish., Ltd (Vietnam), and kept in an indoor aquarium. They were maintained in 350 L of recirculating seawater in a glass tank (L90 cm x W62 cm x H60 cm) under a 12 h light:12 h dark regime, with water conditions and light intensity as described previously (Ip et al. 2015), and acclimatized to laboratory conditions for at least 1 month before experiments. All applicable international, national, and/or institutional (National University of Singapore) guidelines for the care and use of animals were followed.

### Experimental conditions

A batch of *T. squamosa* (*N *=* *5; control) was sampled at the end of the 12 h dark period of the 12 h light:12 h dark regime. Three batches of *T. squamosa* (*N *=* *5 each) were sampled 3, 6, or 12 h after exposure to light. For tissue sampling, giant clams were forced open, and the abductor muscles were cut. The entire right and left ctenidium were dissected out, blotted dry and immediately freeze‐clamped with liquid‐nitrogen‐precooled aluminum tongs. All samples were stored at −80°C until analyzed. Tissues for immunofluorescence microscopy were sampled from *T. squamosa* which had been exposed to darkness (control) or light for 12 h (*N *=* *5 each) followed with anesthetization in 0.2% phenoxyethanol.

### mRNA extraction and cDNA synthesis

The total RNA was extracted from the ctenidium sample using TRI Reagent^®^ (Sigma‐Aldrich Co., St. Louis, MO), and the extracted total RNA was further purified using the RNeasy Plus Mini Kit (Qiagen GmbH, Hilden, Germany). The purified total RNA was quantified using a Shimadzu BioSpec‐nano spectrophotometer (Shimadzu Corporation, Tokyo, Japan) and checked electrophoretically to verify the RNA integrity. The purified total RNA (4 *μ*g) was reverse transcribed into cDNA using a RevertAid^™^ first‐strand cDNA synthesis kit (Thermo Fisher Scientific Inc., Waltham, MA).

### PCR, RACE PCR, and sequencing

The partial *TSNHE3* sequence was obtained using primers (Forward: 5′‐GCVAAATAGGYTTYCATC‐3′ and Reverse: 5′‐CTGYACAAAGACWGTGAAG‐3′) designed according to the conserved regions of *Aplysia californica NHE 3‐like* (XM_005100984.2), *Lingula anatina NHE3‐like* (XM_013560580.1) and *Patiria pectinifera NHE3* (EF514911.1). PCR was carried out in a 9902 Veriti 96‐well thermal cycler (Applied Biosystems, Carlsbad, CA) using DreamTaq^™^ polymerase (Thermo Fisher Scientific Inc.). The cycling conditions were 94°C (3 min), followed by 35 cycles of 94°C (30 sec), 50°C (30 sec), 72°C (2 min) and 1 cycle of final extension at 72°C (10 min). PCR products were separated by electrophoresis in 1% agarose gel. Bands of the estimated size were extracted from the gels using FavorPrep Gel Purification Mini Kit (Favorgen Biotech Corp., Ping‐Tung, Taiwan) according to manufacturer's protocol. PCR products were cloned into pGEM^®^‐T Easy vector (Promega Corporation). The ligated vector was transformed into JM109 competent cells and plated onto Luria‐Bertani (LB) agar with ampicillin, X‐gal and IPTG. Selected white colonies were grown overnight in LB with ampicillin. The plasmids were extracted using the resin‐based plasmid miniprep kit (Axygen Biosciences, Union city, CA). Multiple clones of each fragment were sequenced bidirectionally. The fragments were verified to be *NHE3‐like transporter* from Genbank database. Analysis of multiple clones of *TSNHE3* fragment did not reveal the presence of subisoforms. Specific primers (Forward: 5′‐CAGTTTATCATGGCGTTCGGTGGACTTC‐3′ and Reverse: 5′‐CTCTGAAGTGGTTGCGACATGCGTG‐3′) were designed to obtain the complete cDNA sequence using 5′ and 3′ RACE (SMARTer^™^ RACE cDNA amplification kit: Clontech Laboratories, Mountain View, CA). Multiple sequencing was performed in both directions using BigDye^®^ Terminator v3.1 Cycle Sequencing Kit (Thermo Fisher Scientific Inc.) and subsequently purified by ethanol/sodium acetate precipitation. Purified products were automatically sequenced using the 3130XL Genetic Analyzer (Thermo Fisher Scientific Inc.) to obtain the full‐length cDNA. Sequence assembly and analysis were performed using BioEdit version 7.2.5. The cDNA sequence of *TSNHE3* was deposited into GenBank with the accession number KX685672.

### Deduced amino acid sequence and phylogenetic analysis

The TSNHE3 amino acid sequence was translated from the nucleotide sequence of *TSNHE3* using ExPASy Proteomic server (http://web.expasy.org/translate/). The identity of the deduced amino acid sequence of TSNHE3 was checked by a Blast protein–protein tool (BlastP; http://www.ncbi.nlm.nih.gov/BLAST) with the nonredundant protein database at the National Center for Biotechnology Information (NCBI). The transmembrane domains were predicted using the TMpred program (http://www.ch.embnet.org/software/TMPRED_form.html).

Amino acid sequences of NHE or NHE‐like isoforms from other animals were obtained from Genbank or UniProtKB/TrEMBL (Supporting information Table S1). The sequences were aligned using ClustalX2 and phenogramic analysis was performed to confirm the identity of TSNHE3 using neighbor‐joining method and 100 bootstrap replicates with Phylip.

### Antibodies

The anti‐TSNHE3 antibody was raised as a rabbit polyclonal antibody against residues 573‐586 (LIARSRSPRIDEDE) of TSNHE3 by a commercial firm (GenScript, Piscataway, NJ, USA). The anti‐*α*‐tubulin antibody (12G10) was obtained from the Developmental Studies Hybridoma Bank maintained by the University of Iowa, Department of Biological Sciences, Iowa City, IA 52242.

### Immunofluorescence microscopy

Samples of the ctenidium were excised and immersion fixed overnight in 3% paraformaldehyde in seawater at 4°C. Samples were dehydrated in ethanol and cleared in Histoclear (Sigma‐Aldrich Co.) before embedding in paraffin. The paraffin‐embedded samples were sectioned (3 *μ*m) and collected on slides. Antigen retrieval was performed by treating deparaffinized sections with 1% SDS for 10 min. Sections were blocked using Fast Blocker (Thermo Fisher Scientific Inc.) for 10 min, and subsequently labeled using the custom‐made anti‐TSNHE3 antibody (Genscript). The primary antibody was diluted 1:400 in blocking buffer and incubated at 37°C for 1 h. The secondary antibody incubation was performed at 37°C for 1 h using goat anti‐rabbit Alexa Fluor 488 (1:800 dilution; Life Technologies Corporation). The sections were then rinsed three times with TPBS (0.05% Tween 20 in Phosphate‐buffered saline: 10 mmol L^−1^ Na_2_HPO_4_, 1.8 mmol L^−1^ KH_2_PO_4_, 137 mmol L^−1^ NaCl, 2.7 mmol L^−1^ KCl, pH 7.4), counterstained with DAPI nuclear stain and mounted in ProLong Gold Antifade Mountant (Life Technologies Corporation). A peptide competition assay was performed to validate the specificity of the anti‐TSNHE3 antibody. The anti‐TSNHE3 antibody (25 *μ*g) was incubated with the immunizing peptide (125 *μ*g) provided by GenScript (Piscataway, NJ) in a total volume of 200 *μ*L for 1 h at 25°C. The resulting medium containing the antibody was diluted in Pierce Fast Blocking Buffer and used for immuno‐staining.

An Olympus BX60 epifluorescence microscope (Olympus Corporation, Tokyo, Japan) was used for viewing the sections, and images were captured using the Olympus DP73 digital camera (Olympus Corporation). Optimal exposure settings were predetermined and all images captured under these settings. The corresponding differential interference contrast (DIC) image was also captured for tissue orientation.

Quantifications of total fluorescence intensities were performed on original images captured at 400 ×  magnification for both ctenidial filaments and tertiary water channels of giant clams kept in darkness (control) or exposed to light for 12 h, using Image J version 1.50i software with an Olympus Viewer Plugin (http://rsbweb.nih.gov.libproxy1.nus.edu.sg/ij/). Images were converted to grayscale. A total of five ctenidial filaments and five tertiary water channels were randomly chosen from each image for measurement. For each filament or water channel, six different regions (the summation of which represented at least 50% of the total area) were randomly chosen for measurement. Regions of similar areas adjacent to the apical membrane with little fluorescence were selected for background subtraction. The area, integrated density and mean grey value were used to calculate the total fluorescent intensities in both dark and light samples based on the method of Potapova et al. (2011). Results represent the total fluorescence of five ctenidial filaments or water channels for each individual image. A total of 10 individual images were quantified (*N *=* *5 for control kept in darkness, *N *=* *5 for clams exposed to 12 h of light).

### Quantitative real‐time PCR

RNA (4 *μ*g) from the ctenidia of *T. squamosa* were extracted as mentioned above and reverse‐transcribed using random hexamer primers with RevertAid^TM^ first strand cDNA synthesis kit. qPCR was performed in triplicates using a StepOnePlus^TM^ Real‐Time PCR System (Applied Biosystems). The mRNA expression level of *TSNHE3* was determined using specific forward (5′‐CATAGAGCACAACCTGTCCA ‐3′) and reverse (5′‐ATGTTCCAATCCAGAGTGTCC‐3′) qPCR primers. The qPCR reactions contained 5 *μ*L of 2× Fast SYBR^®^ Green Master Mix (Applied Biosystems), 0.3 *μ*mol L^−1^ of forward and reverse primers each and various quantities of standard (to construct the standard curve) or 1 ng of sample cDNA in a total volume of 10 *μ*L. The cycling conditions, melt curve analysis and construction of a standard curve were performed according to the method of Hiong et al. (2017). The amplification efficiency for *TSNHE3* was 96.3%. The quantity of *TSNHE3* transcripts in a sample was determined from the linear regression line derived from the standard curve and expressed as copy number per ng of total RNA.

### SDS‐PAGE electrophoresis and Western blotting

The tissue samples were homogenized two times in five volumes (w/v) of ice cold buffer containing 50 mmol L^−1^ Tris HCl, (pH 7.4), 1 mmol L^−1^ EDTA, 150 mmol L^−1^ NaCl, 1 mmol L^−1^ NaF, 1 mmol L^−1^ Na_3_VO_4_, 1% NP‐40, 1% sodium deoxycholate, 1 mmol L^−1^ PMSF, and 1× HALT^™^ protease inhibitor cocktail (Thermo Fisher Scientific Inc.) at 50 Hz for 3 min each with 10 sec intervals using the TissueLyser LT (Qiagen GmbH). The homogenate was centrifuged at 10,000*g* for 20 min at 4°C. The protein concentration in the supernatant obtained was determined according to the method of Bradford (1976) and adjusted to 2.5 *μ*g *μ*L^−1^ with Laemmli buffer (Laemmli 1970). Samples were heated at 70°C for 15 min, and then kept at −80°C until analysis.

Fifty micrograms of protein were separated by SDS‐PAGE (8% acrylamide for resolving gel, 4% acrylamide for stacking gel) under conditions as described by Laemmli (1970) using a vertical mini‐slab apparatus (Bio‐Rad Laboratories, Hercules, CA). Proteins were then electrophoretically transferred onto PVDF membranes using a transfer apparatus (Bio‐Rad Laboratories). After transfer, Western blotting was performed using the Pierce Fast Western Blot kit, SuperSignal^®^ West Pico Substrate (Thermo Fisher Scientific Inc.) according to the manufacturer's instructions with slight modifications. Briefly, membranes were incubated with anti‐TSNHE3 antibody (1.25 *μ*g mL^−1^) or anti‐*α*‐tubulin antibody (0.05 *μ*g mL^−1^) for 1 h at 25°C and then incubated in optimized concentrations of anti‐rabbit or anti‐mouse horseradish peroxidase‐conjugated secondary antibodies for 10 min at 25°C. The membranes were washed six times with the Fast Western Wash Buffer provided in the kit in order to have a clear background. Immunoreactive band of TSNHE3 was visualized at the molecular mass of ~80 kDa. Bands were visualized by chemiluminescence provided in the Fast Western Blot kit (Thermo Fisher Scientific Inc.) using X‐ray films (CL‐XPosure^TM^ film, Thermo Fisher Scientific Inc.) which were processed by a Kodak X‐Omat 3000 RA processor (Kodak, Rochester, NY). The developed films were scanned using a CanonScan 9000F Mark II flatbed scanner in TIFF format at 300 dpi resolution. Densitometric quantification of band intensities were performed using ImageJ (version 1.50i, NIH), calibrated with a 37‐step reflection scanner scale (1″ × 8″; Stouffer #R3705‐1C). Results were presented as relative protein abundance of TSNHE3 normalized with *α*‐tubulin.

### Statistical analysis

Results are presented as means ± standard errors of means (SEM.). Statistical analyses were performed using SPSS version 21 (IBM Corporation, Armonk, NY). Homogeneity of variance was checked using Levene's Test. Differences between means were evaluated by one‐way analysis of variance (ANOVA) followed by multiple comparisons of means by Dunnett's T3 (for unequal variance) or by Tukey's test (for equal variance). Differences were regarded as statistically significant at *P *<* *0.05.

## Results

### Nucleotide sequences, translated amino acid sequences and phylogenetic analysis

**Figure 2:**
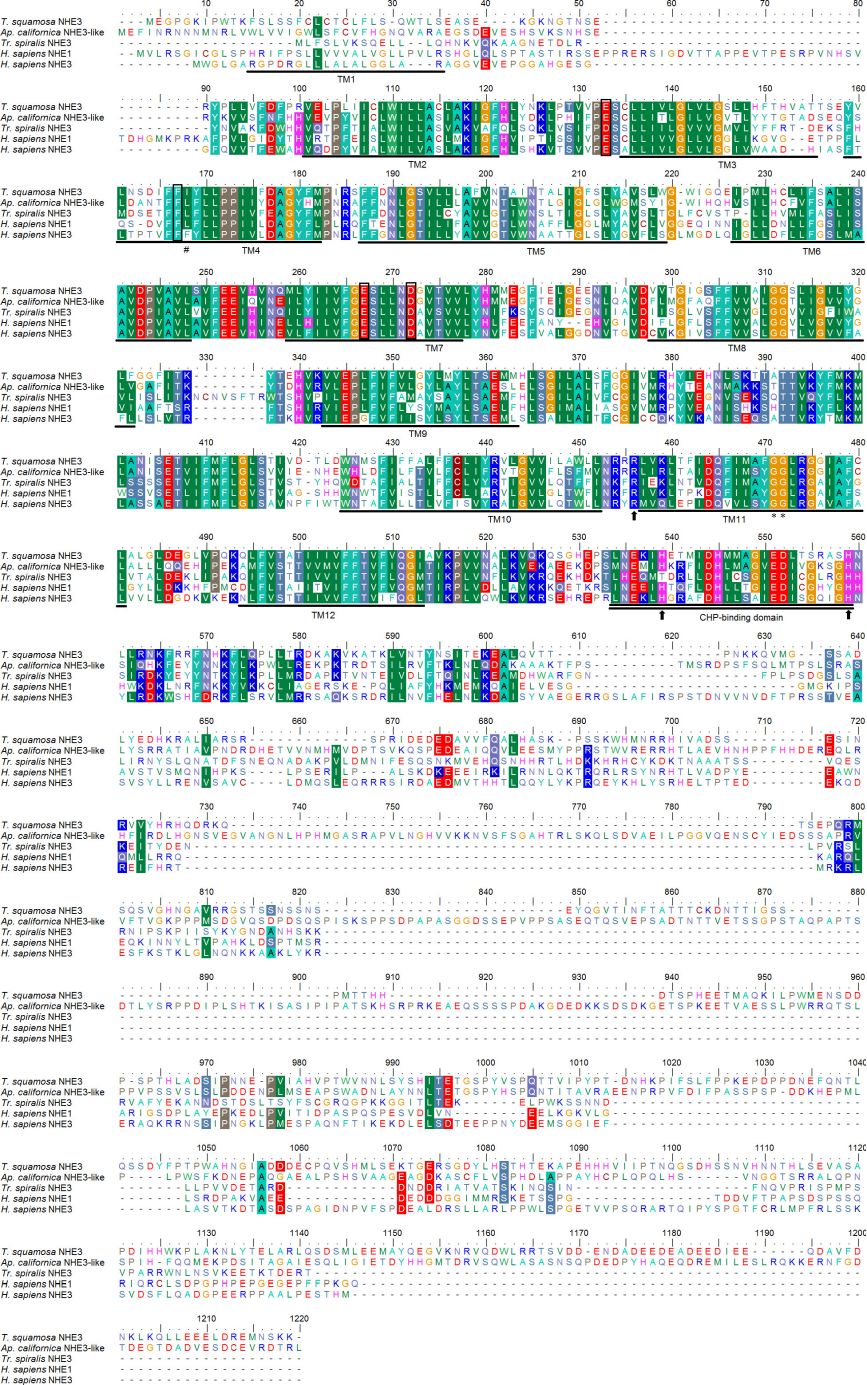
A molecular characterization of Na^+^/H^+^ exchanger 3‐like transporter from the ctenidium of *Tridacna squamosa* (TSNHE3). A multiple amino acid alignment of TSNHE3 from the ctenidium of *T. squamosa*, with four other known NHE or NHE3‐like transporter from *Aplysia californica* (XP_005101041.1), *Trichinella spiralis* (KRY29938.1), *Homo sapiens* NHE1 (NP_003038.2) and *H. sapiens* NHE3 (NP_004165.2). Identical or strongly similar amino acids are indicated by shaded residues. Vertical boxes and asterisks represent important amino acid residues that are involved in Na^+^ and H^+^ binding, respectively. The arrows denote the highly conserved amino acid residues that are important for intracellular pH sensing. The hash marks represent the amino acid residues that are important in determining sensitivity or resistance to amiloride in NHE. The 12 predicted transmembrane regions (TM1‐TM12) and calcineurin homologous protein (CHP) binding domain are underlined and double underlined, respectively. The transmembrane domains of TSNHE3 were predicted using the TMpred program (http://www.ch.embnet.org/software/TMPRED_form.html).

**Figure 3:**
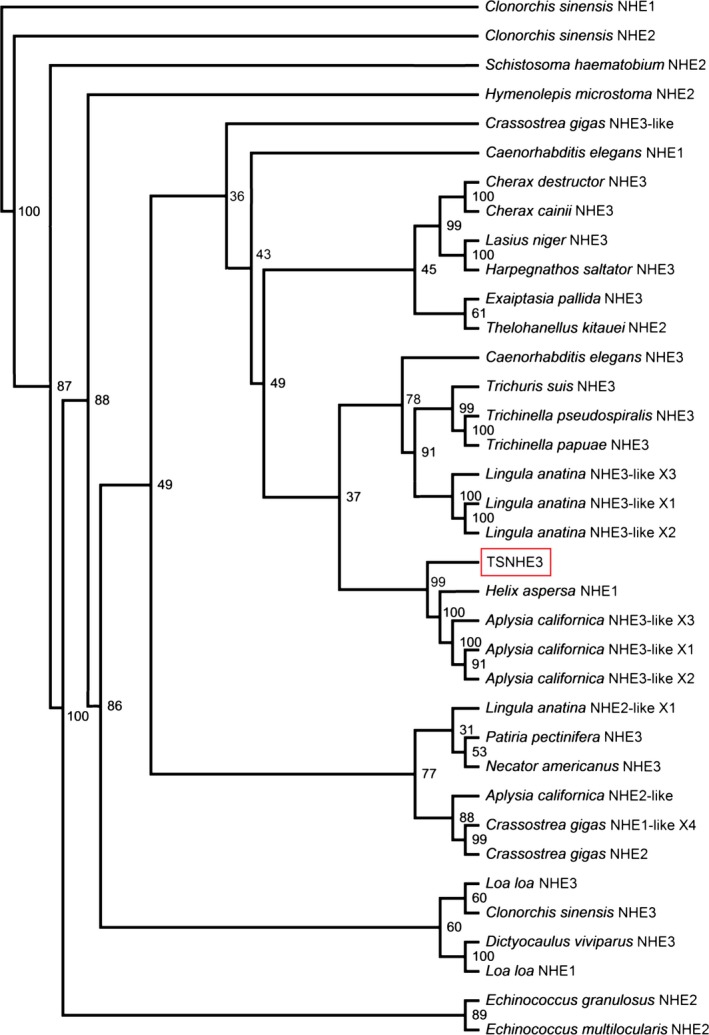
Phenogramic analysis of the Na^+^/H^+^ exchanger 3‐like transporter from the ctenidium of *Tridacna squamosa* (TSNHE3). Numbers presented at each branch point represent bootstrap values from 100 replicates. NHE1 from *Clonorchis sinensis* is used as the outgroup for the phenogramic analysis.

The complete coding sequence of *TSNHE3* from the ctenidium of *T. squamosa* comprised 2886 bp, encoding 961 amino acids with an estimated molecular mass of 105.7 kDa. The top ten results of BlastP using the deduced TSNHE3 of *T. squamosa* as the query revealed that it was similar to the NHE3‐like isoforms of *A. californica* and *L. anatina* (Table 1). The deduced TSNHE3 sequence had 12 predicted transmembrane regions (TM1‐TM12; Fig. 2). Indeed, a phenogramic analysis confirmed that TSNHE3 of *T. squamosa* was closely related to NHE3 or NHE3‐like transporter of other mollusks and nematodes, distinct from other NHE or NHE‐like isoforms (Fig. 3).

**Table 1:** The top 10 results from a protein BLAST (BlastP program, version 2.6.0) of the deduced amino acid sequence of Na^+^/H^+^ exchanger 3‐like transporter from the ctenidia of *Tridacna squamosa* using default settings

No	Description	Accession number	E‐value	Max score (bits)	Total Score	Query cover (%)	Identity (%)
1	PREDICTED: probable Na(+)/H(+) antiporter nhx‐9 [*Crassostrea gigas*]	XP_011424858.1	0	866	866	96	49
2	PREDICTED: sodium/hydrogen exchanger 3‐like isoform X1 [*Aplysia californica*]	XP_005101041.1	0	688	790	89	52
3	PREDICTED: sodium/hydrogen exchanger 3‐like isoform X2 [*Aplysia californica*]	XP_005101042.1	0	649	751	83	53
4	PREDICTED: sodium/hydrogen exchanger 3‐like isoform X3 [*Aplysia californica*]	XP_005101043.1	0	647	749	84	52
5	PREDICTED: sodium/hydrogen exchanger 3‐like isoform X3 [*Lingula anatina*]	XP_013416034.1	0	608	608	74	41
6	PREDICTED: sodium/hydrogen exchanger 3‐like isoform X2 [*Lingula anatina*]	XP_013416033.1	0	607	651	65	54
7	PREDICTED: sodium/hydrogen exchanger 3‐like isoform X1 [*Lingula anatina*]	XP_013416032.1	0	607	650	62	54
8	Sodium/hydrogen exchanger NHE1 [*Helix aspersa*]	AAT35815.1	0	602	723	74	60
9	Na^+^/H^+^ exchanger [*Portunus trituberculatus*]	ANV19765.1	0	599	599	53	54
10	PREDICTED: uncharacterized protein LOC106615301 isoform X7 [*Bactrocera oleae*]	XP_014086946.1	0	597	599	53	54

### Immunofluorescence microscopy

**Figure 4:**
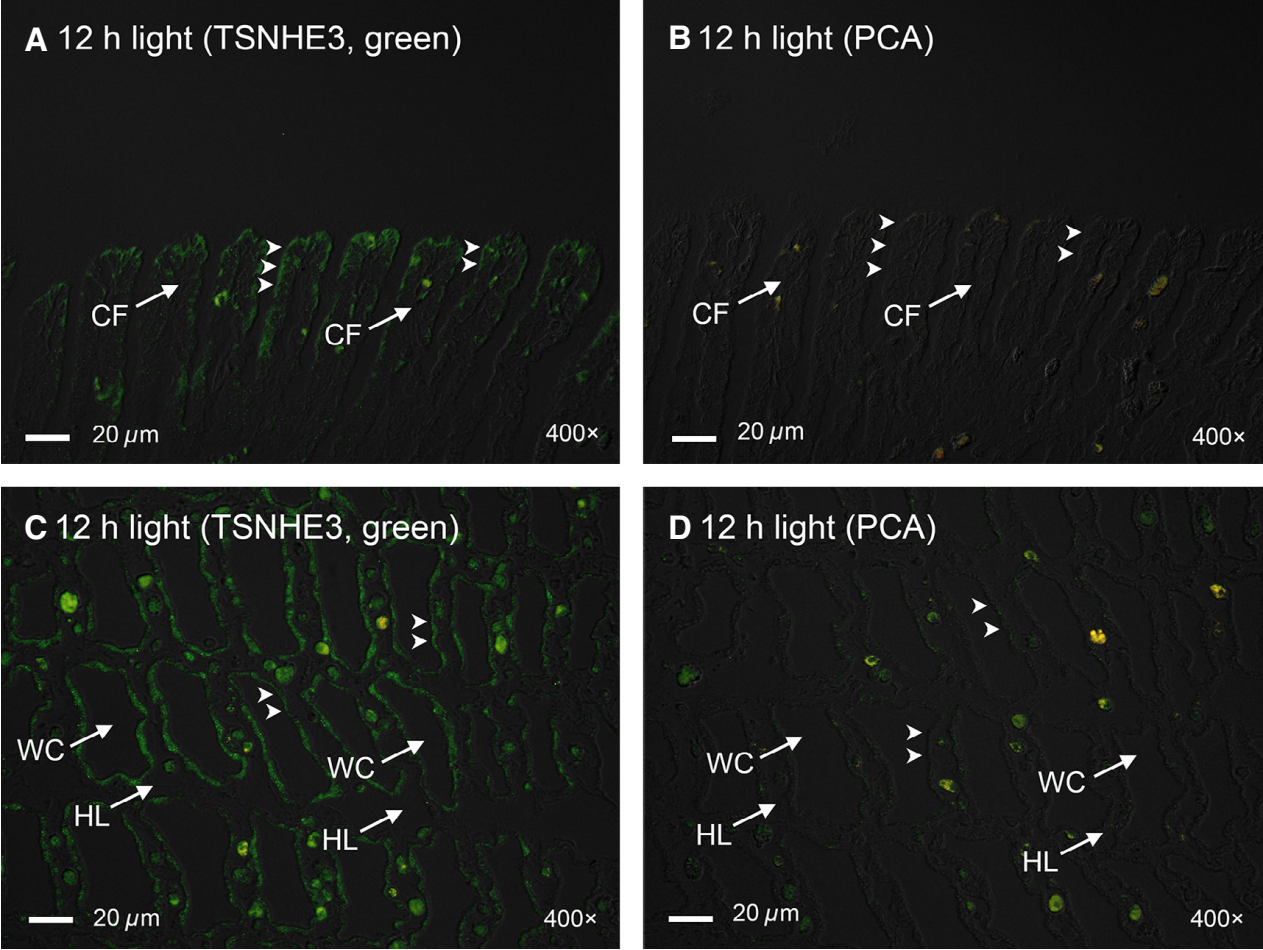
Validation of immunostaining of the Na^+^/H^+^ exchanger 3‐like transporter of *Tridacna squamosa* (TSNHE3) in the ctenidium using a custom‐made anti‐TSNHE3 antibody through a peptide competition assay (PCA). Immunofluorescent localization of TSNHE3 in the ctenidial filaments (CFs) and tertiary water channels (WCs) of a ctenidium of *T. squamosa* exposed to 12 h of light using an anti‐TSNHE3 antibody (A, C), or the same anti‐TSNHE3 antibody pre‐incubated with the immunizing peptide in PCA (B, D). The anti‐TSNHE3 immunofluorescence is shown in green, overlaid with differential interference contrast images. Arrowheads in (A) show TSNHE3‐immunostaining of the apical membrane (close arrowhead) of the epithelial cells in a CF as compared to the lack of TSNHE3 immunostaining with PCA in (B). Arrowheads in (C) show TSNHE3‐immunostaining of the apical membrane (close arrowhead) of the epithelial cells lining a WC as compared to the lack of TSNHE3 immunostaining with PCA in (D). HL, hemolymph. Magnification: 200 ×  for (A, B); 400 ×  for (C, D).

**Figure 5:**
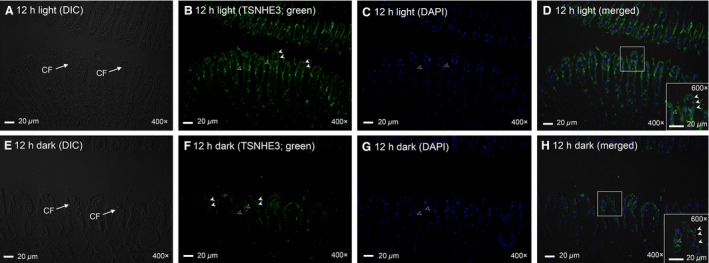
Immunofluorescent localization of the Na^+^/H^+^ exchanger 3‐like transporter of *Tridacna squamosa* (TSNHE3) in the ctenidial filaments (CFs) of the ctenidium. Immunofluorescent localization of TSNHE3 in the CFs of *T. squamosa* exposed to 12 h of light (A–D) or 12 h of darkness (control; E–H). Differential interference contrast images show the structure of CFs (A, E). Anti‐TSNHE3 immunofluorescence is shown in green (B, F) with nuclei counterstained with DAPI in blue (C, G). Green and blue channels are merged and overlaid with the respective differential interference contrast image (D, H). Arrowheads denote TSNHE3‐immunostaining of the apical membrane (close arrowhead) and nucleus (open arrowhead) of the epithelial cells of a CF. Magnification: 200 ×  for (A‐H) with insets of D and H at 400 × . Reproducible results were obtained from five individual clams for each experimental condition.

**Figure 6:**
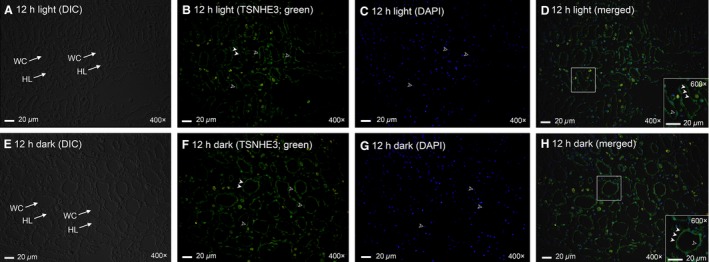
Immunofluorescence localization of the Na^+^/H^+^ exchanger 3‐like transporter of *Tridacna squamosa* (TSNHE3) in the tertiary water channels (WCs) of the ctenidium. Immunofluorescent localization of TSNHE3 in the WCs of the ctenidium of *T. squamosa* exposed to 12 h of light (A–D) or 12 h of darkness (E–H). Differential interference contrast images show the structure and organization of WCs (A, E). Anti‐TSNHE3 immunofluorescence is shown in green (B, F) with nuclei counterstained with DAPI in blue (C, G). Green and blue channels are merged and overlaid with the respective differential interference contrast image (D, H). Arrowheads denote TSNHE3‐immunostaining of the apical membrane (close arrowhead) and nucleus (open arrowhead) of the epithelial cells lining a WC. HL, hemolymph. Magnification: 400 ×  for (A–H) with insets of (D) and (H) at 600 × . Reproducible results were obtained from five individual clams for each experimental condition.

There are two nonpigmented ctenidia in *T. squamosa*, one on each side of the digestive and reproductive mass. Each ctenidium consists of one dorsal (or lateral) and one ventral (or medial) demibranches (Norton and Jones 1992). TSNHE3‐immunostaining was detected at the apical membrane of epithelial cells along the ctenidial branchial filaments of both dorsal and ventral demibranches and around the tertiary water channels of *T. squamosa* (Fig. 4A, C). The validity of TSNHE3‐immunolabeling was verified through a blocking peptide competition test (Fig. 4B, D). Twelve hours of illumination apparently led to an increase in the immunofluorescent TSNHE3‐staining along the apical membrane of the filamentous epithelial cells (Fig. 5B, D) as compared to the control kept in darkness for 12 h (Fig. 5F, H). Indeed, a quantification (integrated density) of immunofluorescence of the apical lining of the filamentous epithelial cells using ImageJ confirmed that the TSNHE3‐immunostaining of the former (2762 ± 185; *N *=* *5) was significantly greater (*P *<* *0.05; ~2‐fold) than that of the latter (1342 ± 448; *N *=* *5). In contrast, the TSNHE3‐immunostaining of the apical membranes of the epithelial cells surrounding the tertiary water channels in the dorsal and ventral demibranches of clams exposed to light (Fig. 6B, D) or kept in darkness (Fig. 6F, H) were comparable. Some nuclei in the dorsal and ventral demibranches of *T. squamosa* were immunoreactive to the custom‐made anti‐TSNHE3 antibody (Fig. 5, 6).

### mRNA expression level and protein abundance

**Figure 7:**
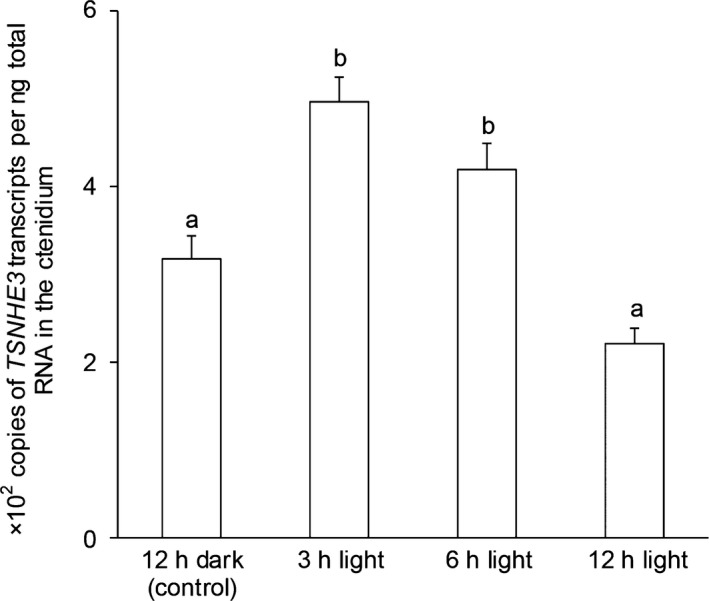
Effects of light on the mRNA expression levels of the *Na* ^*+*^ */H* ^*+*^ *exchanger 3‐like transporter* of *Tridacna squamosa* (*TSNHE3*) in the ctenidium. Absolute quantification (x 10^2^ copies of transcripts per ng of total RNA) of *TSNHE3* transcripts in the ctenidium of *T. squamosa* exposed to 12 h of darkness (control) or 3, 6 or 12 h of light. Results represent means ± SEM (*N *=* *4). Means not sharing the same letter are significantly different (*P *<* *0.05).

**Figure 8:**
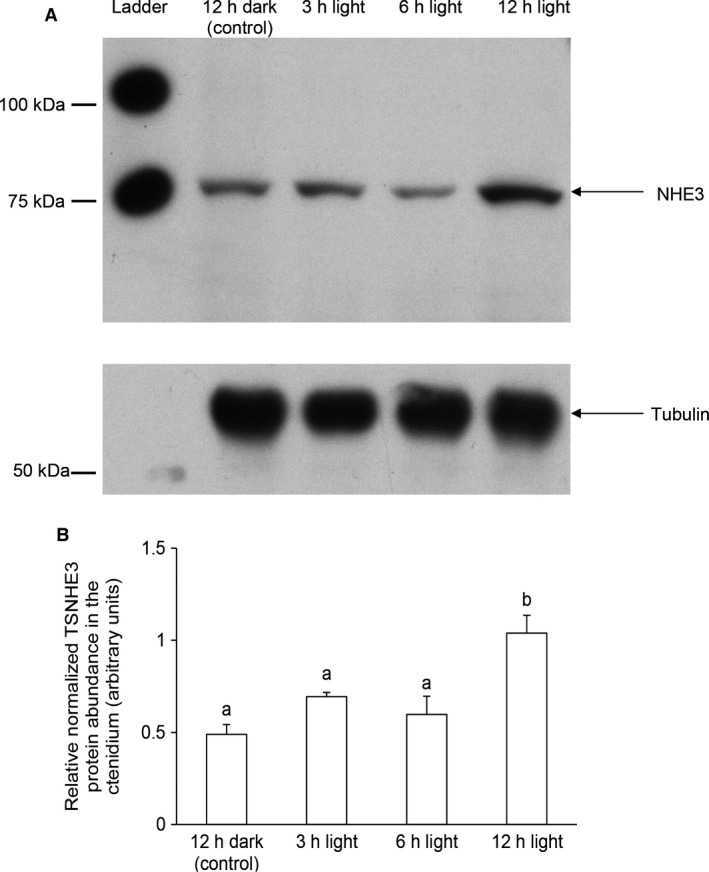
Effects of light on the protein abundances of the Na^+^/H^+^ exchanger 3‐like transporter of *Tridacna squamosa* (TSNHE3) in the ctenidium. Protein abundances of TSNHE3 in the ctenidium of *T. squamosa* exposed to 12 h of darkness (control) or 3, 6 or 12 h of light. (A) An example of immunoblot of TSNHE3 with tubulin as a reference protein. (B) The intensity of the TSNHE3 band for 50 *μ*g protein was normalized with respect to that of tubulin. Results represent means ± SEM (*N *=* *4). Means not sharing the same letter are significantly different (*P *<* *0.05).

There was a significant increase (*P *<* *0.05) in the mRNA expression level of *TSNHE3* in the ctenidium of *T. squamosa* exposed to light for 3 or 6 h (Fig. 7). Western blotting revealed a band of ~80 kDa which was slightly lower than the deduced molecular mass of TSNHE3 (105.7 kDa). Exposure to light for 12 h resulted in a significant increase (*P *<* *0.05) in the protein abundance of ctenidial TSNHE3 (Fig. 8).

## Discussion

### NHE, acid‐base balance, and calcification in bivalves

NHEs are transporters found in virtually all animal species. Being expressed mainly in the plasma membrane, they function primarily to prevent intracellular acidification by catalyzing the electroneutral removal of one intracellular H^+^ in exchange for one extracellular Na^+^ (Orlowski et al. 1992). In particular, NHE3 is expressed in the kidney and gastrointestinal tract of mammals, where it mediates the transepithelial absorption of NaCl and/or NaHCO_3_ (Orlowski et al. 1992; Counillon and Pouyssegur 2000; Donowitz et al. 2013). In kidney, the regulation of NHE3 activity is essential to the maintenance of Na^+^ homeostasis, extracellular fluid volume, blood pressure and acid‐base balance.

Freshwater bivalves are hyperosmotic to the external medium. They have to actively absorb ions through epithelial transporters in order to maintain the high osmolality of the extracellular fluid. In freshwater bivalves, both Na^+^ and Cl^−^ uptake occur through the ctenidium (Dietz and Findley 1980; Dietz and Hagar 1990). Transport kinetic studies involving specific inhibitors indicate the presence of NHE and Cl^−^/HCO_3_^−^ exchanger in ctenidia of freshwater bivalves (Dietz 1978; Dietz and Branton 1979). Intertidal bivalves frequently experience acidosis during air exposure at low tide (Booth et al. 1984; Lindinger et al. 1984), and they have the ability to extrude H^+^ in exchange for Na^+^ uptake through ctenidial NHE during acidosis (Byrne and Dietz 1997). In contrast, marine bivalves are osmoconformers, and there is no evidence of NHE being expressed in their ctenidia. Booth et al. (1984) reported that the marine bivalve, *Mytilus edulis*, had a limited ability to regulate blood pH, and concluded that it did not have to maintain the blood pH in a narrow range due to the lack of pH‐dependent respiratory pigments. However, as shell formation results in the release of H^+^, bivalves, including marine species, must have mechanisms to excrete the excess H^+^ produced during calcification in order to prevent acidosis (Wilbur 1983). Such mechanisms are especially important to giant clams which undergo light‐enhanced calcification leading to high rates of H^+^ production every day during insolation. Here, we report for the first time the expression of *TSNHE3*/TSNHE3 in the ctenidium of *T. squamosa*.

### Molecular characterization of TSNHE3

Like other NHEs, NHE3 has a long C‐terminal cytoplasmic loop and a N‐terminal membrane domain. NHE3 has 11‐13 predicted transmembrane domains for the N‐terminal region (Orlowski et al. 1992; Zizak et al. 2000) and the TSNHE3 of *T. squamosa* has 12. An alignment of the TSNHE3 with NHE isoforms of other species revealed highly conserved residues such as Glu133, Phe167, Glu267, Asp272, and Glu406 (according to the alignment in Fig. 2). These amino acids coordinate the binding of Na^+^ for ion transport (Murtazina et al. 2001; Hisamitsu et al. 2007). The double glycine motif (Gly471/Gly472) in TM11 is important in coordinating the H^+^ binding site (Wakabayashi et al. 2003). Furthermore, Arg456, His539, and His559 are highly conserved and are responsible for intracellular pH sensing (Cha et al. 2003), and are therefore important for the exchanger's conformation changes and activation upon the detection of intracellular pH changes (Wakabayashi et al. 2003). Amiloride is an inhibitor of NHE (Benos 1982) and different NHE isoforms show varying affinity for this inhibitor with the following order of sensitivity: NHE1 ≥  NHE2 >  NHE5 >  NHE3 (Khadilkar et al. 2001). The Leu168 of human NHE1 (according to alignment in Fig. 2) was identified as an important residue involved in binding to amiloride. Mutagenesis of this residue to phenylalanine resulted in an increased resistance to amiloride similar to NHE3 (Slepkov et al. 2007). In TSNHE3, the leucine residue is replaced by an isoleucine and this may affect the binding affinity to amiloride. Other amino acid residues involved in binding to amiloride, including Glu361 and Gly367 (according to alignment in Fig. 2), are conserved in TSNHE3 (Khadilkar et al. 2001). The C‐terminal cytoplasmic domain of NHE3, which can bind to multiple proteins such as CHP, NHERF, CaM kinase II (CaM KII), ezrin, and phospholipase C*γ* (Donowitz and Li 2007), is important for regulating the activity of the exchanger. Indeed, a CHP‐binding domain, which shares high similarity with other animal species, is present in TSNHE3. However, whether other proteins are involved in the regulation of TSNHE3 is unclear at present.

Of note, the molecular mass of TSNHE3 estimated through Western blotting (Fig. 8; 80 kDa) was slightly smaller than the estimated molecular mass based on the deduced amino acid sequence. Nonetheless, this is not uncommon as similar observations have been reported for NHE3 from the kidneys of mouse and rabbit, as well as Nhe3 from the gills of an euryhaline teleost, *Fundulus heteroclitus* (Biemesderfer et al. 1993; Edwards et al. 2005; Li et al. 2013). This phenomenon is common among membrane proteins which display differential binding to SDS resulting in anomalous SDS‐PAGE migration (Rath et al. 2009). Thus, some membrane proteins may move faster in SDS‐PAGE and appear to have a smaller molecular mass than expected (Thornhill and Levinson 1987).

### Subcellular localization of NHE3 in the ctenidia of *T. squamosa*

Immunofluorescence microscopy revealed that TSNHE3 was localized to the apical membrane of epithelial cells along the ctenidial filaments of *T. squamosa*. This is in agreement with the subcellular localization of NHE3 in other types of epithelium. NHE3 is found mainly in the brush border/apical membrane of duodenum, jejunum, ileum, proximal colon, gall bladder, proximal tubule, and thick ascending limb, as well as the proximal portion of the long descending thin limb of Henle (Zachos et al. 2005). In the intestine and proximal tubule, NHE3 operates at the apical membrane to facilitate the absorption of large amounts of Na^+^ through a high capacity and low efficiency process called neutral NaCl absorption (Brett et al. 2005). NHE3 is also expressed in the apical membrane of branchial epithelial cells in teleost fishes (Claiborne et al. 1999; Choe et al. 2002; Edwards et al. 2005). In gills of freshwater teleosts, the apical NHE3 may help to absorb Na^+^ from the external medium, while in gills of marine teleosts, the apical NHE3 may be involved in the excretion of H^+^ for acid‐base balance (Claiborne et al. 1999). Similarly, the apical TSNHE3 expressed in the filamentous epithelial cells of the ctenidium of *T. squamosa* may function to excrete H^+^ in exchange for Na^+^ for acid‐base regulation.

It has been established that certain channels (Bkaily 1994), Ca^2+^‐ATPase (Abernica et al. 2003), Na^+^/K^+^‐ATPase (Garner 2002), Na^+^/Ca^2+^ exchanger (Xie et al. 2002), and NHE (Bkaily et al. 2002) are expressed in the nuclear envelope membranes and nucleoplasm of several types of cells of different animal species. Specifically, NHE of the nuclear envelope membranes can be involved in the regulation of nucleoplasmic pH, and to operate in collaboration with the Na^+^/Ca^2+^ exchanger to facilitate the uptake of cytoplasmic Ca^2+^ by the nucleus (Bkaily et al. 2004). The regulation of peri‐nucleoplasmic and nucleoplasmic pH as well as Ca^2+^ and Na^+^ concentrations may play an important role in modulating nuclear activities such as gene expression, protein synthesis and trafficking of macromolecules through the nuclear pore complex (Bkaily et al. 2006). As some of the nuclei in the ctenidium are TSNHE3‐immunopositive, it is possible that TSNHE3 is involved in regulating nucleoplasmic pH in the ctenidial epithelial cells of *T. squamosa*. Light‐enhanced calcification must be supported by an increase in the uptake of Ca^2+^ from the environment. At present, the sites and mechanisms of increased Ca^2+^ uptake in giant clams is uncertain, but it is possible that increased Ca^2+^ uptake occurs through the ctenidium, and if that is the case, TSNHE3 could be involved in the direct and indirect maintenance of nucleoplasmic pH and Ca^2+^ concentration, respectively. Hence, efforts should be made in the future to examine the possible role of the ctenidium in increased Ca^2+^ uptake in *T. squamosa* exposed to light.

### Light‐dependent expression of TSNHE3/TSNHE3 in the ctenidia of *T. squamosa*

The ctenidium is nonpigmented and contains much lower quantities of symbiotic zooxanthellae as compared with the extensible outer mantle (Ip et al. 2015). A pair of ctenidia is positioned inside the mantle cavity shielded from direct illumination. In general, light‐specific sensors contain pigments that can be oxidized by light, and animal tissues without pigments are usually not light‐responsive. However, our results reveal that the mRNA expression level and protein abundance of *TSNHE3*/TSNHE3 in the ctenidia of *T. squamosa* are light‐dependent, which suggest for the first time that TSNHE3 may play an indirect but important role in light‐enhanced calcification. Light exposure might lead to upregulation of the transcription and translation of *TSNHE3*/TSNHE3. Alternatively, there could be a downregulation in the degradation or turnover of *TSNHE3*/TSNHE3 in response to light. It is possible that light is detected directly by the host clam through its siphonal eyes (Wilkens 1986), which transmit neural signals to the ctenidia. Alternatively, the symbiotic zooxanthellae residing in the extensible outer mantle or the ctenidia may act as “light‐sensing” elements and produce some signaling molecules for the clam host as proposed previously (Ip et al. 2015). These signaling molecules, after being released to the extracellular fluid of the clam host, may activate the transcription and translation of *TSNHE3*/TSNHE3 in the epithelial cells of the ctenidia. Hence, it would be essential to examine the light‐sensing role of zooxanthellae in their symbiotic relationship with the host clam in the future.

The increase in expression of *TSNHE3*/TSNHE3 in the ctenidium can be interpreted as an important light‐dependent response to increase H^+^ excretion, which denotes an important relationship between ctenidial TSNHE3 and light‐enhanced calcification in *T. squamosa*. As calcification leads to the production of one mole of H^+^ for every mole of CaCO_3_ generated in the extrapallial fluid, the adjacent shell‐facing epithelium of the inner mantle must rapidly remove the excess H^+^ during light‐enhanced calcification. Excess H^+^ has to be transported across the shell‐facing epithelium of the inner mantle to the hemolymph in order to avoid intracellularly acidification in these epithelial cells. Our results suggest that the excess H^+^ transported to the hemolymph can be shuttled to and excreted through the ctenidia to achieve whole‐body acid‐base balance. More importantly, they indicate that light‐enhanced calcification in giant clams involves a collaboration between the inner mantle and the ctenidia, which, despite being physically separated, are connected through the hemolymph.

### Perspectives and significance

Several hypotheses have been proposed to explain light‐enhanced calcification in hard corals (Tambutté et al. 2011), and it is assumed that they can also be applied to light‐enhanced calcification in giant clams. For hard corals (Miller and Yellowlees 1989), zooxanthellae are found intracellularly inside symbiosomes in host cells which are adjacent to the coelenteron and close to the calcification site. Hence, Goreau (1959) proposed that H^+^ released during calcification in hard corals could react with HCO_3_^−^ to form CO_2_, and photosynthesis in the symbiotic zooxanthellae could drain CO_2_ from the calcification site and thus favor CaCO_3_ precipitation. In contrast, giant clams harbor extracellular zooxanthellae mainly in the extensible outer mantle which is far away from the site of calcification. If the excess H^+^ produced during light‐enhanced calcification was to react with HCO_3_^−^ in the extrapallial fluid to produce CO_2_, it would reduce the concentration of HCO_3_^−^ therein and lower the solubility product of [Ca^2+^] and [HCO_3_^−^], which would reduce the rate of CaCO_3_ precipitation. Moreover, even if occurred, the CO_2_ produced in the extrapallial fluid would not be readily available to the majority of zooxanthellae residing remotely in the extensible outer mantle. Rather, when taken together with information in the literature, our results indicate that the H^+^ produced in the extrapallial fluid of *T. squamosa* during light‐enhanced calcification can be transported into the inner mantle tentatively through an unknown NH_4_^+^ transporter (Ip et al. 2006, 2015) or plasma membrane Ca^2+^‐ATPase (Sano et al. 2012), and the H^+^ is then shuttled through the hemolymph to the ctenidia where it is excreted by the apical TSNHE3 in exchange for Na^+^. Compared with corals, giant clams have more complex organs/tissues with a considerable degree of division of labor between them. Hence, it is not unexpected that light‐enhanced calcification in *T. squamosa* requires the cooperation between the inner mantle and the ctenidia. More importantly, our results demonstrate that light can affect the expression of a host's transport mechanism engaging indirectly in the calcification process in an organ remote from the calcification site, providing new insights into the mechanisms of light‐enhanced calcification in giant clams.

## Conflict of Interest

No conflicts of interest, financial or otherwise, are declared by the authors.

## Data Accessibility

## References

[phy213209-bib-0001] Abernica, B. , G. N. Pierce , and J. S. Gilchrist . 2003 Nucleoplasmic calcium regulation in rabbit aortic vascular smooth muscle cells. Can. J. Physiol. Pharmacol. 81:301–310. 12733828 10.1139/y03-005

[phy213209-bib-0002] Benos, D. J. 1982 Amiloride: a molecular probe of sodium transport in tissues and cells. Am. J. Physiol. 242:C131–C145. 7039345 10.1152/ajpcell.1982.242.3.C131

[phy213209-bib-0003] Biemesderfer, D. , J. Pizzonia , A. Abu‐Alfa , M. Exner , R. Reilly , P. Igarashi , et al. 1993 NHE3: a Na^+^/H^+^ exchanger isoform of renal brush border. Am. J. Physiol. 265:F736–F742. 8238556 10.1152/ajprenal.1993.265.5.F736

[phy213209-bib-0004] Bkaily, G. 1994 The possible role of Ca^2+^ and K^+^ channels in vascular smooth muscle physiopathology Pp. 103–113 in Bkaily G. R. G., ed. Ionic channels in vascular smooth muscle. TX, Landes Company, Austin.

[phy213209-bib-0005] Bkaily, G. , D. Massaad , S. Choufani , D. Jacques , and P. D'Orleans‐Juste . 2002 Role of endothelin‐1 receptors in the sarcolemma membrane and the nuclear membrane in the modulation of basal cytosolic and nuclear calcium levels in heart cells. Clin. Sci. (Lond.) 103(Suppl. 48):141S–147S. 12193073 10.1042/CS103S141S

[phy213209-bib-0006] Bkaily, G. , M. Nader , L. Avedanian , D. Jacques , C. Perrault , D. Abdel‐Samad , et al. 2004 Immunofluorescence revealed the presence of NHE‐1 in the nuclear membranes of rat cardiomyocytes and isolated nuclei of human, rabbit, and rat aortic and liver tissues. Can. J. Physiol. Pharmacol. 82:805–811. 15523538 10.1139/y04-119

[phy213209-bib-0007] Bkaily, G. , M. Nader , L. Avedanian , S. Choufani , D. Jacques , P. D'Orléans‐Juste , et al. 2006 G‐protein‐coupled receptors, channels, and Na^+^‐H^+^ exchanger in nuclear membranes of heart, hepatic, vascular endothelial, and smooth muscle cells. Can. J. Physiol. Pharmacol. 84:431–441. 16902588 10.1139/y06-002

[phy213209-bib-0008] Booth, C. E. , D. G. McDonald , and P. J. Walsh . 1984 Acid‐base balance in the sea mussel, *Mytilus edulis*. I. Effects of hypoxia and air‐exposure on hemolymph acid‐base status. Mar. Biol. Lett. 5:347–358.

[phy213209-bib-0009] Bradford, M. M. 1976 Rapid and sensitive method for quantitation of microgram quantities of protein utilizing the principle of protein‐dye binding. Anal. Biochem. 72:248–254. 942051 10.1016/0003-2697(76)90527-3

[phy213209-bib-0010] Brett, C. L. , D. N. Tukaye , S. Mukherjee , and R. Rao . 2005 The yeast endosomal Na^+^K^+^H^+^ exchanger Nhx1 regulates cellular pH to control vesicle trafficking. Mol. Biol. Cell 16:1396–1405. 15635088 10.1091/mbc.E04-11-0999 PMC551501

[phy213209-bib-0011] Byrne, R. A. , and T. H. Dietz . 1997 Ion transport and acid‐base balance in freshwater bivalves. J. Exp. Biol. 200:457–465. 9318114 10.1242/jeb.200.3.457

[phy213209-bib-0012] Cha, B. , S. Oh , J. Shanmugaratnam , M. Donowitz , and C. C. Yun . 2003 Two histidine residues in the juxta‐membrane cytoplasmic domain of Na^+^/H^+^ exchanger isoform 3 (NHE3) determine the set point. J. Membr. Biol. 191:49–58. 12532276 10.1007/s00232-002-1044-2

[phy213209-bib-0013] Choe, K. P. , A. I. Morrison‐Shetlar , B. P. Wall , and J. B. Claiborne . 2002 Immunological detection of Na^+^/H^+^ exchangers in the gills of hagfish, *Myxine glutinosa*, an elasmobranch, *Raja erinacea*, and a teleost *Fundulus heteroclitus* . Comp. Biochem. Physiol. 131A:375–385. 10.1016/s1095-6433(01)00491-3 11818226

[phy213209-bib-0014] Choi, J. Y. , M. Shah , M. G. Lee , P. J. Schultheis , G. E. Shull , S. Muallem , et al. 2000 Novel amiloride‐sensitive sodium‐dependent proton secretion in the mouse proximal convoluted tubule. J. Clin. Invest. 105:1141–1146. 10772659 10.1172/JCI9260 PMC300838

[phy213209-bib-0015] Claiborne, J. B. , C. R. Blackston , K. P. Choe , D. C. Dawson , S. P. Harris , L. A. Mackenzie , et al. 1999 A mechanism for branchial acid excretion in marine fish: identification of multiple Na^+^/H^+^ antiporter (NHE) isoforms in gills of two seawater teleosts. J. Exp. Biol. 202:315–324. 9882643 10.1242/jeb.202.3.315

[phy213209-bib-0016] Counillon, L. , and J. Pouyssegur . 2000 The expanding family of eucaryotic Na^+^/H^+^ exchangers. J. Biol. Chem. 275:1–4. 10617577 10.1074/jbc.275.1.1

[phy213209-bib-0017] Dietz, T. H. 1978 Na transport in the freshwater mussel, *Carunculina texasensis* (Lea). Am. J. Physiol. 235:R35–R40. 677338 10.1152/ajpregu.1978.235.1.R35

[phy213209-bib-0018] Dietz, T. H. , and W. D. Branton . 1979 Cl^−^ transport in freshwater mussels. Physiol. Zool. 52:520–528.

[phy213209-bib-0019] Dietz, T. H. , and A. M. Findley . 1980 Ion‐stimulated ATPase activity and NaCl uptake in the gills of freshwater mussels. Can. J. Zool. 58:917–923.

[phy213209-bib-0020] Dietz, T. H. , and A. F. Hagar . 1990 Cl uptake in isolated gills of the freshwater mussel *Ligumia subrostrata* . Can. J. Zool. 68:6–9.

[phy213209-bib-0021] Donowitz, M. , and X. Li . 2007 Regulatory binding partners and complexes of NHE3. Physiol. Rev. 87:825–887. 17615390 10.1152/physrev.00030.2006

[phy213209-bib-0022] Donowitz, M. , and M. Tse . 2001 Molecular physiology of mammalian epithelial Na^+^/H^+^ exchangers NHE2 and NHE3 Pp. 437–498 *in* Barrett K. E., Donowitz M., eds. Gastrointestinal Transport Molecular Physiology: Current Topics in Membranes, Vol. 50. Academic Press, San Diego, CA.

[phy213209-bib-0023] Donowitz, M. , C. Ming Tse , D. Fuster **.** 2013 SLC9/NHE gene family, a plasma membrane and organellar family of Na^+^/H^+^ exchangers. Mol. Aspects Med. 34:236–251. 23506868 10.1016/j.mam.2012.05.001 PMC3724465

[phy213209-bib-0024] Edwards, S. L. , B. P. Wall , A. I. Morrison‐Shetlar , S. Sligh , J. C. Weakley , and J. B. Claiborne . 2005 The effect of environmental hypercapnia and salinity on the expression of NHE‐like isoforms in the gills of a euryhaline fish (*Fundulus heteroclitus*). J. Exp. Zool. A Comp. Exp. Biol. 303:464–475. 15880778 10.1002/jez.a.175

[phy213209-bib-0025] Fliegel, L. , and P. Dibrov . 1996 Biochemistry and molecular biology of the Na^+^/H^+^ exchanger: an overview Pp. 1–20 *in* Fliegel L. ed. The Na^+^/H^+^ exchanger, Springer/ R. G. Landes Company, Austin, TX.

[phy213209-bib-0026] Garner, M. H. 2002 Na, K‐ATPase in the nuclear envelope regulates Na^+^: K^+^ gradients in hepatocyte nuclei. J. Membr. Biol. 187:97–115. 12029368 10.1007/s00232-001-0155-5

[phy213209-bib-0027] Goreau, T. F. 1959 The physiology of skeleton formation in corals. I. A method for measuring the rate of calcium deposition by corals under different conditions. Biol. Bull. 116:59–75.

[phy213209-bib-0028] Griffiths, D. J. , H. Winsor , and T. Luongvan . 1992 Iridophores in the mantle of giant clams. Aust. J. Zool. 40:319–326.

[phy213209-bib-0029] Hiong, K. C. , C. Y. L. Choo , M. V. Boo , B. Ching , W. P. Wong , S. F. Chew , et al. 2017 A light‐dependent ammonia‐assimilating mechanism in the ctenidia of a giant clam. Coral Reefs. 36:311–323.

[phy213209-bib-0030] Hisamitsu, T. , K. Yamada , T. Y. Nakamura , and S. Wakabayashi . 2007 Functional importance of charged residues within the putative intracellular loops in pH regulation by Na^+^/H^+^ exchanger NHE1. FEBS J. 274:4326–4335. 17662110 10.1111/j.1742-4658.2007.05962.x

[phy213209-bib-0031] Holt, A. L. , S. Vahidinia , Y. L. Gagnon , D. E. Morse , and A. M. Sweeney . 2014 Photosymbiotic giant clams are transformers of solar flux. J. R. Soc. Interface 11:20140678. doi:(10.1098/rsif.2014.0678). 25401182 10.1098/rsif.2014.0678 PMC4223897

[phy213209-bib-0032] Hu, M. Y. , Y. C. Tseng , M. Stumpp , M. A. Gutowska , R. Kiko , M. Lucassen , et al. 2011 Elevated seawater P_CO2_ differentially affects branchial acid‐base transporters over the course of development in the cephalopod *Sepia officinalis* . Am. J. Physiol. Regul. Integr. Comp. Physiol. 300:R1100–R1114. 21307359 10.1152/ajpregu.00653.2010

[phy213209-bib-0033] Hu, M. Y. , Y. J. Guh , M. Stumpp , J. R. Lee , R. D. Chen , P. H. Sung , et al. 2014 Branchial NH_4_ ^+^‐dependent acid‐base transport mechanisms and energy metabolism of squid (*Sepioteuthis lessoniana*) affected by seawater acidification. Front. Zool. 11:55. doi:10.1186/s12983‐014‐0055‐z.

[phy213209-bib-0034] Hwang, P. P. , and T. H. Lee . 2007 New insights into fish ion regulation and mitochondrion‐rich cells. Comp. Biochem. Physiol. A Mol. Integr. Physiol. 48:479–497. 10.1016/j.cbpa.2007.06.416 17689996

[phy213209-bib-0035] Ip, Y. K. , A. M. Loong , K. C. Hiong , W. P. Wong , S. F. Chew , K. Reddy , et al. 2006 Light induces an increase in the pH of and a decrease in the ammonia concentration in the extrapallial fluid of the giant clam *Tridacna squamosa* . Physiol. Biochem. Zool. 79:656–664. 16691530 10.1086/501061

[phy213209-bib-0036] Ip, Y. K. , B. Ching , K. C. Hiong , C. Y. L. Choo , M. V. Boo , W. P. Wong , et al. 2015 Light induces changes in activities of Na^+^/K^+^‐ATPase, H^+^/K^+^‐ATPase and glutamine synthetase in tissues involved directly or indirectly in light‐enhanced calcification in the giant clam *Tridacna squamosa* . Front. Physiol. 6:68. doi:10.3389/fphys.2015.00068. 25798110 10.3389/fphys.2015.00068 PMC4351588

[phy213209-bib-0037] Khadilkar, A. , P. Iannuzzi , and J. Orlowski . 2001 Identification of sites in the second exomembrane loop and ninth transmembrane helix of the mammalian Na^+^/H^+^ exchanger important for drug recognition and cation translocation. J. Biol. Chem. 276:43792–43800. 11564737 10.1074/jbc.M106659200

[phy213209-bib-0038] Laemmli, U. K. 1970 Cleavage of structural proteins during the assembly of the head of bacteriophage T4. Nature 227:680–685. 5432063 10.1038/227680a0

[phy213209-bib-0039] Li, H. C. , Z. Du , S. Barone , I. Rubera , A. A. McDonough , M. Tauc , et al. 2013 Proximal tubule specific knockout of the Na^+^/H^+^ exchanger NHE3: effects on bicarbonate absorption and ammonium excretion. J. Mol. Med. 91:951–963. 23508938 10.1007/s00109-013-1015-3 PMC3730089

[phy213209-bib-0040] Lindinger, M. I. , D. J. Lauren , and D. G. McDonald . 1984 Acid‐base balance in the sea mussel, *Mytilus edulis*. III. Effects of environmental hypercapnia on intra‐ and extracellular acid‐base balance. Mar. Biol. Lett. 5:371–381.

[phy213209-bib-0041] Lucas, J. S. , W. J. Nash , C. M. Crawford , and R. D. Braley . 1989 Growth and survival during the ocean‐nursery rearing of giant clams *Tridacna gigas* . Aquaculture 80:45–61.

[phy213209-bib-0042] Miller, D. J. , and D. Yellowlees . 1989 Inorganic nitrogen uptake by symbiotic marine cnidarians: a critical review. Proc. R. Soc. Lond. Ser. B 237:109–125.

[phy213209-bib-0043] Murtazina, R. , B. J. Booth , B. L. Bullis , D. N. Singh , and L. Fliegel . 2001 Functional analysis of polar amino‐acid residues in membrane‐associated regions of the NHE1 isoform of the mammalian Na^+^/H^+^ exchanger. Eur. J. Biochem. 268:4674–4685. 11532004 10.1046/j.1432-1327.2001.02391.x

[phy213209-bib-0044] Norton, J. H. , and G. W. Jones . 1992 The giant clam: an anatomical and histological atlas. Australian Centre for International Agricultural Research, Canberra, Australia.

[phy213209-bib-0045] Norton, J. H. , M. A. Shepherd , H. M. Long , and W. K. Fitt . 1992 The zooxanthellae tubular system in the giant clam. Biol. Bull. 183:503–506. 10.2307/1542028 29300506

[phy213209-bib-0046] Orlowski, J. , R. A. Kandasamy , and G. E. Shull . 1992 Molecular cloning of putative members of the Na/H exchanger gene family. cDNA cloning, deduced amino acid sequence, and mRNA tissue expression of the rat Na/H exchanger NHE‐1 and two structurally related proteins. J. Biol. Chem. 267:9331–9339. 1577762

[phy213209-bib-0047] Potapova, T. A. , S. Sivakumar , J. N. Flynn , R. Li , and G. J. Gorbsky . 2011 Mitotic progression becomes irreversible in prometaphase and collapses when Wee1 and Cdc25 are inhibited. Mol. Biol. Cell 22:1191–1206. 21325631 10.1091/mbc.E10-07-0599 PMC3078080

[phy213209-bib-0048] Rath, A. , M. Glibowicka , V. G. Nadeau , G. Chen , and C. M. Deber . 2009 Detergent binding explains anomalous SDS‐PAGE migration of membrane proteins. Proc. Natl Acad. Sci. USA 106:1760–1765. 19181854 10.1073/pnas.0813167106 PMC2644111

[phy213209-bib-0049] Rosewater, J . 1965 The family Tridacnidae in the Indo‐Pacific Indo‐Pac. Mollusca 1:347–396.

[phy213209-bib-0050] Salvador, J. M. , G. Inesi , J. L. Rigaud , and A. M. Mata . 1988 Ca^2+^ transport by reconstituted synaptosomal ATPase is associated with H^+^ countertransport and net charge displacement. J. Biol. Chem. 273:18230–18234. 10.1074/jbc.273.29.18230 9660785

[phy213209-bib-0051] Sano, Y. , S. Kobayashi , K. Shirai , N. Takahata , K. Matsumoto , T. Watanabe , et al. 2012 Past daily light cycle recorded in the strontium/calcium ratios of giant clam shells. Nat. Commun. 3:761. doi:(10.1038/ncomms1763). 22453834 10.1038/ncomms1763

[phy213209-bib-0052] Slepkov, E. R. , J. K. Rainey , B. D. Sykes , and L. Fliegel . 2007 Structural and functional analysis of the Na^+^/H^+^ exchanger. Biochem. J. 401:623–633. 17209804 10.1042/BJ20061062 PMC1770851

[phy213209-bib-0053] Tambutté, S. , M. Holcomb , C. Ferrier‐Pagès , S. Reynaud , E. Tambutté , D. Zoccola , et al. 2011 Coral biomineralization: from the gene to the environment. J. Exp. Mar. Biol. Ecol. 408:58–78.

[phy213209-bib-0054] Thornhill, W. B. , and S. R. Levinson . 1987 Biosynthesis of electroplax sodium channels in *Electrophorus* electrocytes and *Xenopus* oocytes. Biochemistry 26:4381–4388. 2444249 10.1021/bi00388a029

[phy213209-bib-0055] Wakabayashi, S. , T. Hisamitsu , T. Pang , and M. Shigekawa . 2003 Mutations of Arg440 and Gly455/Gly456 oppositely change pH sensing of Na^+^/H^+^ exchanger 1. J. Biol. Chem. 278:11828–11835. 12562776 10.1074/jbc.M213243200

[phy213209-bib-0056] Wilbur, K. M. 1983 Shell formation Pp. 253–287 *in* Saleuddin A. S. M. and Wilbur K. M., eds. The Mollusca Vol. 4 Academic Press, London, UK.

[phy213209-bib-0057] Wilkens, L. A. 1986 Primary inhibition by light: a unique property of bivalve photoreceptors. Am. Malacol. Bull. 26:101–109.

[phy213209-bib-0058] Xie, X. , G. Wu , Z. H. Lu , and R. W. Ledeen . 2002 Potentiation of a sodium‐calcium exchanger in the nuclear envelope by nuclear GM1 ganglioside. J. Neurochem. 81:1185–1195. 12068067 10.1046/j.1471-4159.2002.00917.x

[phy213209-bib-0059] Zachos, N. C. , M. Tse , and M. Donowitz . 2005 Molecular physiology of intestinal Na^+^/H^+^ exchange. Annu. Rev. Physiol. 67:411–443. 15709964 10.1146/annurev.physiol.67.031103.153004

[phy213209-bib-0060] Zizak, M. , M. E. Cavet , D. Bayle , C. M. Tse , S. Hallen , G. Sachs , et al. 2000 Na^+^/H^+^ exchanger NHE3 has 11 membrane spanning domains and a cleaved signal peptide: topology analysis using in vitro transcription/translation. Biochemistry 39:8102–8112. 10891093 10.1021/bi000870t

